# Diet and Other Modifiable Factors in Long-Term Decline of Kidney Function: Observational and Population-Based Cohort Study

**DOI:** 10.3390/nu15204337

**Published:** 2023-10-11

**Authors:** Massimo Cirillo, Giancarlo Bilancio, Carmine Secondulfo, Oscar Terradura-Vagnarelli, Antonio Pisani, Eleonora Riccio, Martino Laurenzi

**Affiliations:** 1 Department “Scuola Medica Salernitana”, Università di Salerno, 84081 Baronissi, Italy; gbilancio@unisa.it (G.B.); csecondulfo@unisa.it (C.S.); 2 Centro Studi Epidemiologici di Gubbio, 06024 Gubbio, Italy; oscar.terradura@gmail.com (O.T.-V.); mlaurenzi@comcast.net (M.L.); 3 Department Sanità Pubblica, Università di Napoli, 80131 Napoli, Italy; antonio.pisani13@gmail.com; 4 Institute for Biomedical Research and Innovation, National Research Council of Italy, 90146 Palermo, Italy; elyriccio@libero.it

**Keywords:** aging, eGFR, physical activity, alcohol intake, protein, sodium, potassium

## Abstract

Background: Lower physical activity, lower alcohol intake, higher protein intake, higher sodium intake, and lower potassium intake related to greater kidney function decline over time, according to previous studies. The present study aimed to analyze the cumulative effects of these factors. Methods: This prospective, observational, population-based cohort study included 3039 adult examinees of the Gubbio study who participated in the baseline exam and 15-year follow-up exam. Kidney function was evaluated as estimated glomerular filtration rate (eGFR). Habitual physical activity in leisure time and habitual alcohol intake were assessed by questionnaires; dietary intakes of protein, sodium, and potassium were assessed by urinary markers. Based on previous reports, each one of the five modifiable factors was scored 0 for the tertile associated with smaller eGFR decline (low risk), 2 for the tertile associated with greater eGFR decline (high risk), and 1 for the intermediate tertile (intermediate risk). A cumulative score was calculated as the sum of the factor-specific scores and used as the main independent variable. Results: The cumulative score ranged from 0 to 10, that is, from low risk for all factors to high risk for all factors (skewness = 0.032, mean ± SD = 5 ± 2). To avoid the bias of low-n analyses, score 0 was re-coded as 1 and score 10 was recoded as 9; after re-coding, the cumulative score ranged from 1 to 9 (skewness = 0.016, mean ± SD = 5 ± 2). The cumulative score related to annualized eGFR change in multi-variable linear regression (slope = −0.027, 95%CI = −0.039/−0.014, *p* < 0.001); findings were consistent in apparently healthy examinees and other subgroups. De novo incidence of eGFR < 60 mL/min × 1.73 m^2^ was higher along the cumulative score (*p* < 0.001). Compared to score 1 (n examinees = 35, adjusted incidence = 2.0%), incidence of low kidney function was 4.5 times higher in score 5 (n examinees = 624, adjusted incidence = 8.9%) and 6.5 times higher in score 9 (n examinees = 86, adjusted incidence = 12.9%). The cumulative score related to incidence of low kidney function in multi-variable logistic regression (odds ratio = 1.19, 95%CI = 1.08/1.32, *p* < 0.001). Conclusions: The combination of five modifiable factors predicted large differences in long-term incidence of low kidney function.

## 1. Introduction

Low kidney function is a progressive disorder affecting millions of people, and it incurs high social and economic costs due to the related excess risk of disability, dialysis or renal transplantation, and mortality [1,2,3]. It is generally accepted that most cases of low kidney function are due to diabetes and hypertension, although several other causes exist. Low kidney function is more prevalent in older ages without a definite cause [1,2,3]. It is debated whether a decline in kidney function with aging is a physiological phenomenon or not [4]. Although the influence of modifiable factors is often regarded as limited, several studies have reported that dietary habits and other modifiable factors may modulate the decline of kidney function over time [5,6]. In agreement with those studies [5,6], a lower physical activity in leisure time, a lower alcohol intake, a higher protein intake, a higher sodium intake, and a lower potassium intake in the Gubbio study population associated with a greater kidney function, decline over time independently of each other [7,8,9,10]. The Gubbio study is a prospective, longitudinal project investigating cardiovascular disease, kidney disease, and other public health objectives in a sample of the general population residing in a city of north–central Italy [11,12]. The present re-analysis of data collected in the Gubbio study sample of the general population was designed with the aim to quantify the cumulative effects of physical activity, alcohol intake, protein intake, sodium intake, and potassium intake on the long-term decline in kidney function.

## 2. Methods

The Gubbio study is an observational, longitudinal project, ongoing since 1983, on a whole sample of the Italian general population residing in Gubbio, a city in northern–central Italy [11,12]. The study adhered to the Declaration of Helsinki and included informed consent and the approval by the institutional committee (CEAS-Umbria #2850/16). The study design, the invited population, the response rates, and the characteristics of the study cohort were reported [11,12]. The main analyses of the present study dealt with data collected in all individuals with age ≥18 years at the 1989–1992 exam (baseline) and at the 15-year follow-up (follow-up). At both exams the study protocol included the collection of biological samples, the administration of standardized questionnaires by trained personnel, and a medical visit with measurements of anthropometry, heart rate, and blood pressure [11,12]. After-baseline mortality data were collected from local sections of the national registry [12]. Regarding biological samples, the baseline exam also comprised a timed overnight urine collection under fed conditions from the first void after the evening meal to the first void at the morning wake-up, and an early morning blood sample collected under fasting conditions after the completion of the overnight urine collection; the follow-up exam included the collection of an early morning blood sample after an overnight fast [12]. Lab variables were measured on fresh samples using automated biochemistry and quality controls; serum creatinine was measured on frozen samples by automated biochemistry using a kinetic alkaline picrate assay with IDMS-traceable standardization [13]. Regarding the medical visit, blood pressure was measured on the right arm using mercury sphygmomanometers and cuffs of appropriate size in participants that had been seated quietly for 5 min; three measurements were taken one minute apart, and the mean of the second and third measurement was used for analyses [11,12].

### 2.1. Assessment of the Five Modifiable Factors

The baseline and the follow-up questionnaires included specific questions on the habitual physical activity in leisure time expressed as min/d and on the habitual intake of four types of alcohol containing beverages: wine, beer, aperitifs/cocktails, and spirits [11,12]; the total intake of alcohol as g/d was calculated as the rounded sum of the alcohol intake due to the four types of beverages using the Italian standards of volume and alcoholic gradation of each beverage [8]. Urinary levels of urea, sodium, and potassium in the baseline overnight collections were measured as indices of the dietary intake of protein [9,14,15], sodium [7,14,16], and potassium [10,14,17], respectively. Urinary levels of urea, sodium, and potassium were expressed as ratio to urine creatinine for exclusion of errors in completeness, and timing of urine collection and were analyzed separately by gender and age strata to reduce the confounding of gender and age on urinary creatinine [16,17,18].

The reliability of the assessments of the five modifiable factors was assessed in the Supplementary Materials by analyses on specific proxies for each factor: heart rate for physical activity [19], serum gamma-glutamyl-transferase for alcohol intake [20], serum urea for urinary urea as index of protein intake [21], blood hematocrit for urinary sodium as index of sodium intake [22], and serum potassium for urinary potassium as an index of potassium intake [23]. Ancillary analyses in the Supplementary Materials dealt with the consistency over time of the assessment of the modifiable factors as follows: the consistency of physical activity and alcohol intake over time was evaluated in examinees who participated both in the baseline exam and in the follow-up exam; the consistency of urinary dietary markers over time and over different types of urine collection was evaluated in the subgroups of examinees who participated in the baseline exam and had previously participated in the INTERSALT study [14].

### 2.2. Cumulative Score

The cumulative score was a priori conceived to obtain a single numerical variable ranging from 0 to 10 that reflected the cumulative and graded prevalence of the five modifiable factors in each examinee. To this aim, each one of the five modifiable factors was scored from 0 to 2 based on the linear relationships previously found in the Gubbio cohort between quantiles of the given factor and the eGFR change over time [7,8,9,10]. In particular, given that higher urinary urea and higher urinary sodium related to a greater eGFR decline [7,9], urinary urea and urinary sodium were scored 0 when the value was in the lowest tertile, 1 when in the intermediate tertile, and 2 when in the highest tertile (low risk, intermediate risk, and high risk, respectively); vice versa, given that lower physical activity, lower alcohol intake, and lower urinary potassium related to a greater eGFR decline [7,8,10], physical activity, alcohol intake, and urinary potassium were scored 0 when the value was in the highest tertile, 1 when in the intermediate tertile, and 2 when in the lowest tertile. After that, the cumulative score was calculated as the sum of the five factor-specific scores and the study cohort was stratified into eleven groups with scores ranging from 0 to 10, i.e., from low risk for all five factors to high risk for all five factors.

### 2.3. Dependent Variables

The main dependent variables of the study were the baseline to follow-up change in kidney function and the baseline to follow-up de novo incidence of low kidney function. Kidney function was assessed as eGFR calculated by the Chronic Kidney Disease—Epidemiology Collaboration equation using gender, age, and serum creatinine [24,25]. Low kidney function was defined as eGFR < 60 mL/min × 1.73 m^2^ [5]. The absolute eGFR change was calculated as the follow-up eGFR minus the baseline eGFR; the annualized eGFR change was calculated as the absolute eGFR change divided by the time interval between the baseline exam and the follow-up exam; incidence of low kidney function was defined as follow-up eGFR < 60 mL/min × 1.73 m^2^ in examinees with baseline eGFR ≥ 60 mL/min × 1.73 m^2^ [5].

### 2.4. Other Variables

Data in analyses included the dates of the baseline visit and of the follow-up visit, gender, and the baseline data of the followings: age, nonfat mass calculated on the basis of triceps and subscapular skinfold thickness as reported and used as an index of skeletal muscle mass [26]; 24-h urinary creatinine was not measured but estimated by Chronic Kidney Disease—Epidemiology Collaboration equation [18] for two scopes: as an index of creatinine generation from the muscle mass [27] and for estimation of daily intake of protein, sodium, and potassium (see below *Calculations and Statistics*); creatinine concentration measured in overnight urine for expression of urinary dietary markers as ratio to creatinine; smoking status; obesity defined as body mass index ≥30 kg/m^2^; hypertension defined as systolic pressure ≥140 mmHg or diastolic pressure ≥90 mmHg or regular antihypertensive drug treatment; diabetes defined as serum glucose ≥7.0 mmol/L or regular antidiabetic drug treatment; hyperuricemia defined as serum uric acid ≥416 µmol/L or regular hypouricemic drug treatment; history of cardiovascular disease defined as the report of previous diagnoses of myocardial infarction or stroke or heart failure. Albumin in overnight urine was measured only in the subgroup with baseline age 45–64 years and expressed as albumin/creatinine ratio; albuminuria was defined as albumin/creatinine ratio ≥30 mg/g [5]. Examinees were defined as apparently healthy if they were without low kidney function, hypertension, diabetes, hyperuricemia, and history of cardiovascular disease. The incidence of hypertension and the incidence of diabetes were defined as the presence of the disorder at the follow-up exam in examinees without the disorder at the baseline exam.

### 2.5. Calculations and Statistics

Descriptive data were reported as prevalence for categorical variables, mean ± SD for non-skewed numerical variables, and median with interquartile range (IQR) for skewed variables (skewness > 1). Estimates of daily intake of protein, sodium, and potassium were reported for comparability to other studies and calculated as follows: regarding protein intake, the urinary urea/creatinine ratio as g/g was first converted to the urinary urea nitrogen/creatinine ratio as g/g (multiplier = 0.467), then converted to 24-h urinary urea nitrogen as g/d (multiplier = 24-h urinary creatinine), and finally converted to protein intake as g/d (multiplier = 6.25) [15]; the sodium/creatinine ratio and the potassium/creatinine ratio as mmol/g were multiplied by the 24-h urinary creatinine to estimate sodium intake and potassium intake as mmol/d [16,17].

Preliminary statistical procedures investigated whether the cumulative score related to mortality after baseline exam, using multivariable Cox regression. The time for this analysis was calculated as the interval between the baseline exam and the date of death or, in alive examinees, between the baseline exam and the completion of the follow-up period. The list of covariates in the Cox regression model on mortality included sex and baseline data of age, low eGFR, smoking, obesity, hypertension, diabetes, hyperuricemia, and history of cardiovascular disease. Results of Cox regression were reported as hazard ratio (HR) with 95% confidence interval (95%CI).

The main analyses of the study investigated the relation of the cumulative score with the eGFR change from the baseline exam to the follow-up exam (ANOVA with mean centered covariates and linear regression both for absolute eGFR change and for annualized eGFR change) and with the incidence of low kidney function from the baseline exam to the follow-up exam (chi-square analysis, ANOVA, and logistic regression). Separate analyses on the relation of each modifiable factor to kidney function were not included in the present study because they had previously been reported [7,8,9,10]. Covariates in the multi-variable models included gender and the baseline data on age, eGFR, 24-h urinary creatinine, smoking, obesity, hypertension, diabetes, hyperuricemia, and cardiovascular disease. The multi-variable models on absolute eGFR change and on incident low kidney function also included in the covariates list the duration of follow-up, calculated as the time interval from the baseline exam to the follow-up exam. For the sensitivity and consistency analyses, multi-variable models were also investigated in apparently healthy examinees and other subgroups defined by demography, kidney function, alcohol intake, smoking, obesity, hypertension, diabetes, cardiovascular disease history, hyperuricemia, and albuminuria. The results of linear regression were reported as coefficient (slope) in analyses for the whole study cohort and as standardized coefficient (beta) in subgroups analyses to reduce the effect of differences in the distribution of the covariates. The results of the logistic regression were reported as odds ratio (OR). An additional multi-variable logistic model also included in the covariates the incidence of hypertension and the incidence of diabetes given that these disorders are considered major determinants of kidney function decline [1,2,3]. Statistical procedures were performed using IBM-SPSS Statistics version 19 software (IBM, Armonk, NY, USA).

## 3. Results

### 3.1. Descriptive Statistics

Of the 4699 examinees with age ≥18 years at baseline, 4669 had complete data at baseline and 3039 had complete data at baseline and follow-up (Descriptive statistics in Table 1). The mortality-corrected participation in the follow-up exam was 79.0% given that 822 examinees had died prior to the follow-up exam (median follow-up = 16.8 years, IQR = 15.8/17.7, persons × years product = 72,918; annual mortality rate = 1.13%). Table S1 in the Supplementary Materials reported the descriptive statistics at the follow-up exam.

At the baseline exam, nonfat mass (skewness = 0.42; mean ± SD = 50.1 ± 10.2 kg) was strongly correlated with estimated 24-h urinary creatinine (r = 0.936, *p* < 0.001), and positively correlated with serum creatinine (r = 0.286, *p* < 0.001). Table S2 of the Supplementary Materials reported descriptive statistics at baseline of the proxies used to validate the assessments of the modifiable factors.

The mean ± SD was 14.2 ± 2.2 years for the interval between the baseline exam and the follow-up exam, −10.3 ± 10.3 mL/min × 1.73 m^2^ for the absolute eGFR change, and −0.755 ± 0.753 mL/min × 1.73 m^2^ for the annualized eGFR change. In the 3000 examinees with complete data at the baseline and follow-up exams and without low kidney function at the baseline exam, the incidence at the follow-up exam was 8.2% for low kidney function (n = 245, persons × years product = 42,564; annual incidence = 0.58%), 27.9% for hypertension (n = 838, annual incidence = 1.97%), and 5.4% for diabetes (n = 163, annual incidence = 0.38%).

### 3.2. Tertiles of the Modifiable Factors and Factor-Specific Scores

Tables S3–S7 reported descriptive statistics of the tertiles used for the definition of factor-specific scores and the data on the associations with proxy variables. Significant associations were found between higher physical activity and lower heart rate (Table S3), between higher alcohol intake and higher serum gamma-glutamyl-transferase (Table S4), between higher urinary urea and higher serum urea (Table S5), between higher urinary sodium and lower hematocrit (Table S6), and between higher urinary potassium and higher serum potassium (Table S7). Tables S8–S12 showed data on the consistency over time of the habitual physical activity in leisure time, the habitual alcohol intake, and the urinary levels of urea, sodium, and potassium. In examinees who participated both in the baseline exam and in the follow-up exam, significant associations were found between baseline data and follow-up data for physical activity and alcohol intake (Tables S8 and S9). In the subgroups who participated both in the Gubbio study and in the Intersalt study [14], significant associations were found between the overnight collection of the baseline exam and the 24-h collection of the INTERSALT study for urinary urea, urinary sodium, and urinary potassium (Tables S10–S12).

### 3.3. Cumulative Score

The cumulative score ranged from 0 to 10, i.e., from low risk for all factors to high risk for all factors (skewness = 0.032, mean ± SD = 5 ± 2; upper panel of Figure S1 in the Supplementary Materials). To avoid the bias of low-n analyses, the seven examinees with score 0 were merged into the examinees with score 1 and the 19 examinees with score 10 were merged into the examinees with score 9. After the recoding of these low-n tails, the cumulative score ranged from 1 to 9 (skewness = 0.016, mean ± SD = 5 ± 2; lower panel of Figure S1). Findings were similar in the examinees participating both in the baseline exam and the follow-up exam (Figure S2 of the Supplementary Materials).

As expected per definition, the cumulative score inversely associated with physical activity, alcohol intake, and urinary potassium, while it was directly associated with urinary urea nitrogen and urinary sodium (Table 2). Trends along the cumulative score were consistent for the variables used as proxy of the single modifiable factors (Table S13).

### 3.4. Cumulative Score and Mortality Rate

The cumulative score did not relate to mortality in multi-variable Cox regression models (HR = 1.01, 95%CI = 0.97/1.06, *p* = 0.613; covariates list in Methods). Significant predictors of higher mortality were male gender, higher age, low kidney function, smoking, obesity, hypertension, diabetes, and cardiovascular disease (*p* ≤ 0.026).

### 3.5. Cumulative Score and eGFR Change over Time

**Figure 1:**
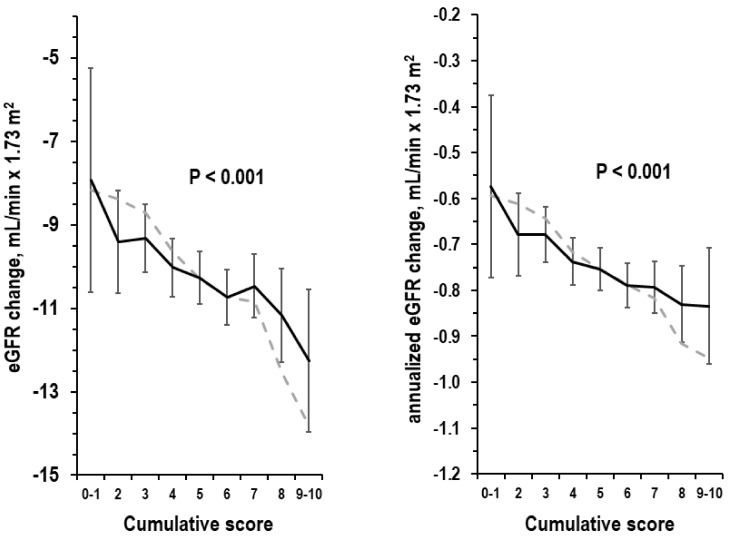
eGFR change from the baseline exam to the follow-up exam by cumulative score. Left panel: absolute eGFR change; right panel: annualized eGFR change. Grey dotted line: non-adjusted mean; black continuous line: adjusted mean with 95%CI. Adjusted ANOVA on absolute eGFR change was controlled for the following covariates: duration of the time interval from baseline exam to follow-up exam, gender, and baseline data of age, eGFR, 24-h urinary creatinine, smoking, obesity, hypertension, diabetes, hyperuricemia, and cardiovascular disease; adjusted ANOVA on annualized eGFR change excluded from the covariates list the duration of the time interval from baseline exam to follow-up exam. *p*-values are for trend by ANOVA with the cumulative score used as a factor; number of examinees from score 0–1 to score 9–10 = 35, 171, 384, 523, 631, 575, 431, 203, and 86.

**Figure 2:**
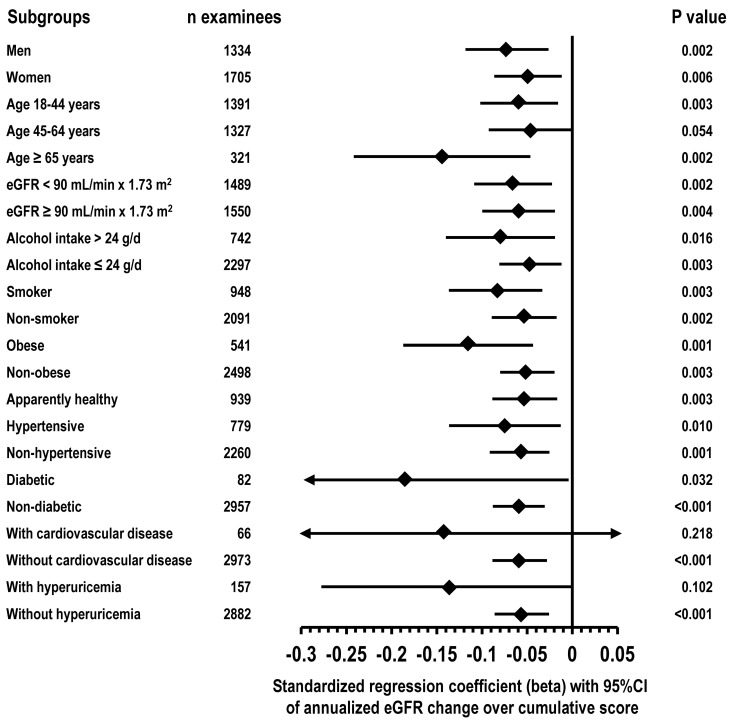
Subgroup multi-variable linear regression of the annualized eGFR change from the baseline exam to the follow-up exam over the cumulative score: type of subgroup, number of examinees, standardized coefficient (beta), 95%CI, and *p*-values. The annualized eGFR change was calculated as the follow-up eGFR minus the baseline eGFR divided by the duration of the time interval from the baseline exam to the follow-up exam. Covariates in the multi-variable regression: gender, and baseline data of age, eGFR, 24-h urinary creatinine, smoking, obesity, hypertension, diabetes, hyperuricemia, and cardiovascular disease.

An inverse trend was found between the cumulative score and the absolute or annualized eGFR change, without and with adjustment for covariates (Figure 1). In multi-variable linear regression, the relation of the cumulative score to the annualized eGFR change was negative (slope = −0.027, 95%CI = −0.039/−0.014; beta = −0.063, 95%CI = −0.092/−0.034, *p* < 0.001; covariates list in Methods). Other independent predictors of more negative annualized eGFR change were female gender, older age, higher eGFR, higher 24-h urinary creatinine, hypertension, and diabetes (*p* ≤ 0.040). In subgroup analyses (Figure 2), beta was consistently negative in both sexes; ages 18–44, 45–64, and ≥65 years; examinees with eGFR < 90 mL/min × 1.73 m^2^ and examinees with eGFR ≥ 90 mL/min × 1.73 mL/min × 1.73 m^2^; examinees with alcohol intake > 24 g/d and examinees with alcohol intake ≤ 24 g/d; smokers and non-smokers; obese and non-obese examinees; apparently healthy examinees; hypertensives and non-hypertensive examinees; diabetics and non-diabetic examinees; with cardiovascular disease and without cardiovascular disease; with hyperuricemia and without hyperuricemia. In the subgroup of examinees with baseline age 45–64 years, the beta of the cumulative score was identical without control for albuminuria and with control for albuminuria (beta = −0.046, 95%CI = −0.093/0.001, *p* = 0.054).

### 3.6. Cumulative Score and Incidence of Low Kidney Function

**Figure 3:**
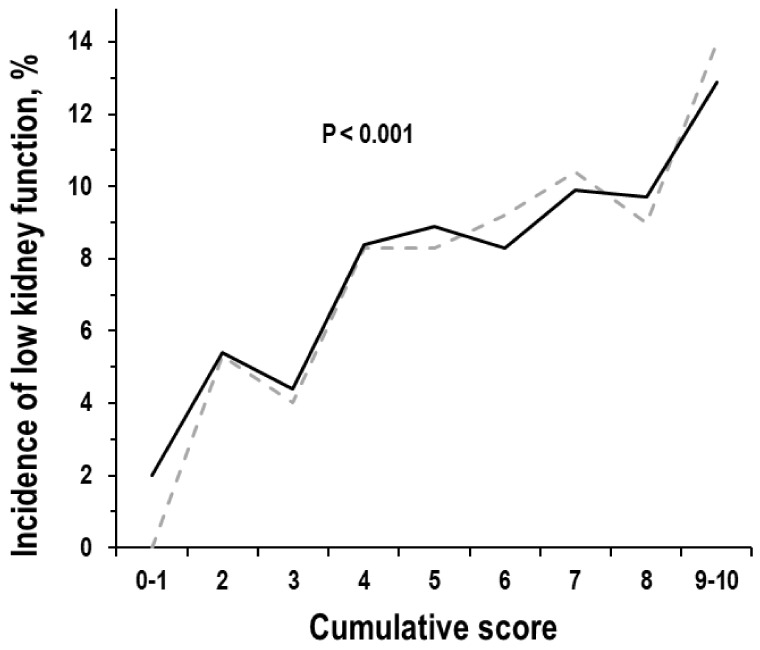
Incidence of low kidney function from the baseline exam to the follow-up exam by the cumulative score: non-adjusted data and adjusted data (grey dotted line and black continuous line). Covariates in multi-variable analysis: duration of time interval from baseline exam to follow-up exam, gender, and baseline data of age, eGFR, 24-h urinary creatinine, physical activity, obesity, smoking, hypertension, diabetes, hyperuricemia, and history of cardiovascular disease. Low kidney function was defined as eGFR < 60 mL/min × 1.73 m^2^. Number of examinees by cumulative score: 35, 170, 378, 518, 624, 567, 422, 200, and 86.

The cumulative score associated with a positive trend in the incidence of low kidney function without adjustment and with adjustment for covariates (Figure 3). Compared to examinees with score 1 (n = 35, adjusted incidence = 2.0%), incidence of low kidney function was 4.5 times higher in examinees with score 5 (n = 624, adjusted incidence = 8.9%) and 6.5 times higher in examinees with score 9 (n = 86, adjusted incidence = 12.9%). A higher cumulative score independently related to a higher incidence of low kidney function in multi-variable logistic regression (OR = 1.19, 95%CI = 1.08/1.32, *p* < 0.001; covariates list in Methods); other significant predictors of the incidence of low kidney function were female gender, older age, and lower eGFR (*p* ≤ 0.029). Findings were also identical in regression controlling for incident hypertension and incident diabetes (OR = 1.19, 95%CI = 1.08/1.32, *p* < 0.001) and were similar in regression limited to the subgroup of apparently heathy examinees (n = 2100; OR = 1.25, 95%CI = 1.06/1.46, *p* = 0.007).

## 4. Discussion

This observational, population-based, long-term study reported the novel finding of a graded and continuous relationship with eGFR change and with incidence of low kidney function of a cumulative score reflecting the combination of the following five modifiable factors: physical activity in leisure time, alcohol intake, protein intake, sodium intake, and potassium intake. The findings were independent of several covariates including classical risk factors for kidney function such as hypertension, diabetes, and albuminuria. The findings were consistent in subgroup analyses and significant also from a clinical viewpoint considering the observed differences in the incidence of low kidney function.

Main study limitations were the use of a single overnight urine collection for the assessment of dietary markers, the dated collection of data, and the lack of information on other dietary factors such as vegetal and animal proteins, carbohydrates, and fats. Regarding the use of a single overnight urine collection, previous data has indicated that, at the individual level, this method implies the risk of misclassification and of regression dilution bias due to the day-to-day variability in the diet of individuals on an unrestricted diet and/or to the circadian variations in kidney functions [14,16,17]. Data of the present study indicated that the misclassification was minor at the level of groups; in fact, the trends along tertiles of urinary markers were consistent in the Gubbio cohort over exams performed at different times and with the use of different types of urine collection (Tables S8–S12 of the Supplementary Materials). The view that the overnight urine collection could give reliable information on the diet composition was supported by the results of the proxy variable measured in morning blood samples withdrawn after the urine collection. It was unlikely that a higher overnight urinary excretion of urea could be followed by a higher serum urea in the morning in the absence of a higher urea generation due to higher protein intake (Table S5) [21]. The same holds true for potassium; a higher potassium intake appeared as the most reasonable explanation of higher overnight urinary potassium given that the higher urinary excretion night-time was followed by a higher serum potassium in the morning (Table S7) [23]. Finally, a similar point could be made for sodium; in the absence of a higher sodium intake, a higher overnight urinary excretion of sodium was expected to be followed by a morning trend to hemoconcentration rather than by a trend to hemodilution (Table S6) [22]. Regarding the dated data collection, the effect of this limitation should be minor considering the stability in the last 25 years of the prevalence of kidney dysfunction in Italy [28] and the parallel stability over a similar period of the associations between modifiable factors and kidney function in the Gubbio study cohort [7,8,9,10]. Regarding the lack of information on other dietary factors, the role of carbohydrates and fats cannot be excluded, although previous data has suggested a minor effect of these nutrients on kidney function, if any [29,30]. On the other hand, the merits of this study include the preliminary analysis on a possible association of the cumulative score with mortality, the analyses on variables used for validation of reported information on physical activity and alcohol intake, and the inclusion of 24-h urinary creatinine in the covariates list of multi-variable analyses as a predictable confounder in the use of eGFR as kidney function index [25].

The results of the present study were in accordance with the conclusions of the meta-analysis of Kelly et al. that analyzed categorical definitions for physical activity, alcohol intake, protein intake, sodium intake, and potassium intake [6]. Given that wine accounted for more than 94% of alcohol intake in the Gubbio cohort [8], it is uncertain if the effects of alcohol could be shared by beverages other than wine. Study results did not confirm the reported association of smoking with kidney function decline over time [6]. A possible explanation for this discrepancy could be that, in this long-term analysis, the smoking-associated mortality excess competed with and diluted the unfavorable effect of smoking on kidney function decline.

Various mechanisms were hypothesized to explain the link between the single modifiable factors and the kidney function decline. The list of these mechanisms included kidney hyperfiltration, natriuresis modulation, anti-vasopressin effects, endothelial protection, and cardiovascular damage [7,8,9,10,29,31,32,33,34,35,36]. Although a population-based, observational study can hardly investigate the mechanism(s) of associations, three observations indicated that the relation of the cumulative score with the change in kidney function over time was not dependent on the baseline kidney function: first, the results were statistically independent of baseline eGFR in multi-variable analyses; second, the results were consistent both for the examinees with baseline eGFR < 90 mL/min × 1.73 m^2^ and for the examinees with baseline eGFR > 90 mL/min × 1.73 m^2^; third, the results were coherent both for eGFR decline over time and for incidence of low kidney function, although a greater eGFR decline was predicted by a higher baseline eGFR while a higher incidence of low kidney function was predicted by a lower baseline eGFR. The relation between the cumulative score and the kidney function change was independent of cardiovascular risk factors and cardiovascular disease and also appeared consistent in subgroup analyses for healthy individuals and young adults. Last, given that the relation was also consistent in the analysis limited to individuals with alcohol intake ≤ 24 g/d, it could not be accounted for by the high alcohol intake associated with high mortality [8,37].

Regarding practical implications, the results of the present study proved that the combination of five modifiable factors in analysis, per se, was a major predictor of the decline of the kidney function associated with aging. Therefore, the study results suggested two conclusions: first, the aging-associated decline in kidney function is not a totally physiologic phenomenon; second, modifiable factors play a pivotal role in the primary prevention of low kidney function. However, the extrapolation of these conclusions to the progression of kidney failure in chronic kidney disease should be applied cautiously due to the low prevalence of this disorder in the study cohort.

Briefly, this observational cohort study reported that, in an Italian sample of the adult general population, a cumulative score reflecting the combination of five modifiable factors was a strong and independent predictor of the long-term decline of kidney function and of the de novo incidence of low kidney function. Therefore, the results suggested that lifestyles are pivotal determinants of the aging-associated decline in kidney function and of the high prevalence of low kidney function in older ages. In particular, the present results indicated that the role of lifestyles appeared strong in individuals with the exposure to an unfavorable profile for the combination of low physical activity in leisure time, no wine intake, high protein intake, high sodium intake, and low potassium intake.

## Figures and Tables

**Table 1:** Descriptive statistics at the baseline exam in the 4669 examinees with complete data at baseline and in the 3039 examinees with complete data at baseline and follow-up. Prevalence for categorical variables, mean ± SD for numerical non-skewed variables, and median (IQR) for numerical skewed variables.

	With Baseline Data	With Baseline Data and Follow-Up Data
Examinees, n Men, %, n Age, years age 18–44 age 45–64 * age ≥ 65	4669 45.1% (2108) 50.0 ± 17.9 38.7% (1805) 36.7% (1714) 24.6% (1150)	3039 43.9% (1334) 45.4 ± 14.7 45.8% (1391) 43.7% (1327) 10.6% (321)
**Modifiable factors**		
Habitual physical activity in leisure time, min/d Habitual alcohol intake, g/d Urea to creatinine ratio in overnight urine, g/g estimated protein intake, g/d Sodium to creatinine ratio in overnight urine, mmol/g estimated sodium intake, mmol/d Potassium to creatinine ratio in overnight urine, mmol/g estimated potassium intake, mmol/d	6 (0/15) 12 (0/24) 16 (12/20) 57 (44/72) 106 (69/153) 127 (84/184) 26 (19/35) 31 (23/42)	6 (0/17) 12 (0/24) 16 (12/20) 57 (45/72) 101 (66/143) 124 (83/174) 24 (18/33) 30 (22/40)
**Kidney function**		
Serum creatinine, µmol/L eGFR, mL/min × 1.73 m^2^ eGFR < 60 mL/min × 1.73 m^2^, % (n)	79 (72/87) 87 ± 17 5.7% (267)	78 (71/86) 90 ± 15 1.3% (39)
**Covariates**		
Estimated 24-h urinary creatinine, g/d Obesity, % (n) Smoking, % (n) Hypertension, % (n) Diabetes, % (n) Hyperuricemia, % (n) Cardiovascular disease, % (n)	1.24 ± 0.32 20.2% (942) 29.4% (1373) 34.3% (1601) 5.3% (248) 7.0% (329) 5.1% (240)	1.27 ± 0.32 17.8% (541) 31.2% (948) 25.6% (779) 2.7% (82) 5.2% (157) 2.2% (66)

* with measured urinary albumin; albuminuria, % (n) = 4.0% (68) in the 1714 examinees with baseline data; 3.5% (46) in the 1327 examinees with baseline data and follow-up data.

**Table 2:** Means of modifiable factors at the baseline exam by cumulative score.

Score	N Examinees	Physical Activity, min/d	Alcohol Intake, g/d	Urinary Urea/ Creatinine, g/g	Urinary Sodium/ Creatinine, mmol/g	Urinary Potassium/ Creatinine, mmol/g
0–1	58	50.9	47.8	10.7	62	31
2	240	35.3	44.9	11.6	73	31
3	564	29.8	37.0	13.2	86	29
4	834	21.7	29.6	14.7	101	29
5	999	16.9	23.5	17.1	113	29
6	903	10.8	15.1	19.2	139	30
7	641	7.3	7.9	21.1	152	29
8	306	5.1	2.5	22.8	173	29
9–10	124	0.8	0.8	24.2	177	22
*p-Value for trend* *along cumulative score* *		*<0.001*	*<0.001*	*<0.001*	*<0.001*	*0.019*

* by ANOVA.

## Data Availability

Data are stored in the Gubbio study repository. For information, write to mlaurenzi@comcast.net.

## Supplementary Materials

The following supporting information can be downloaded at: https://www.mdpi.com/article/10.3390/nu15204337/s1. The Supplementary Materials include descriptive data for the whole cohort, for tertiles of the five modifiable factors, and for subgroups defined by the cumulative score.
