# Supplementary material for: Diet and Other Modifiable Factors in Long-Term Decline of Kidney Function: Observational and Population-Based Cohort Study
Source: Nutrients. 2023 Oct 11;15(20):4337. doi: 10.3390/nu15204337 (PMC10609413; doi:10.3390/nu15204337)
Supplement: Supplementary file 1 [file nutrients-15-04337-s001.zip › nutrients-2638504-supplementary.pdf]

Supplementary

Materials

Table S1 – Descriptive statistics at the follow-up exam in the 3039 examinees with complete data (43.9% men): prevalence for categorical variables, mean±SD for numerical non-skewed variables, and median (IQR) for numerical skewed variables.

	Data at the follow-up exam
Age, years	59.5±13.6
Habitual physical activity in leisure time, min/d	20 (0/40)
Habitual alcohol intake, g/d	6 (0/24)
Overnight urine urea/creatinine ratio, g/g	n.a.
Overnight urine sodium/creatinine ratio, mmol/g	n.a.
Overnight urine potassium/creatinine ratio, mmol/g	n.a.
Serum creatinine, µmol/L	80 (73/87)
eGFR, mL/min x 1.73 m^2	80.1±15.3
Low kidney function, %	9.0%
Estimated 24-hour urinary creatinine, g/d	1.21±0.34
Obesity, %	21.7%
Smoking, %	21.7%
Hypertension, %	51.8%
Diabetes, %	7.9%
Hyperuricemia, %	n.a.
Cardiovascular disease, %	7.8%

n.a. = not available

Table S2 – Descriptive statistics at the baseline exam of the variables used for validation of the assessments of the modifiable factors: mean±SD for numerical non-skewed variables and median (IQR) for numerical skewed variables.

Number of examinees	4669
Heart rate, b/min	71.5±10.4
Serum gamma-glutamyl-transferase, U/L	22 (15/35)
Serum urea, mmol/L	5.83 (5.00/6.83)
Hematocrit, %	42.7±3.9
Serum potassium, mmol/L	4.13±0.37

Table S3 – Tertiles of habitual physical activity in leisure time at the baseline exam: number of examinees, median of physical activity, factor-specific score, gender prevalence, mean age, and mean of heart rate used as the validation variable.

	Tertile of physical activity			*P for trend**
	1	2	3	
Tertiles thresholds, habitual physical activity in leisure time at the baseline exam, min/d	< 6	6 – 15	> 15	
Number of examinees	1992	1625	1052	
Median of habitual physical activity in leisure time at the baseline exam, min/d	0	15	45	
Factor-specific score	2	1	0	
% men	37.1%	40.1%	68.3%	*<0.001*
Age, years	51.6	53.6	41.4	*< 0.001*
Heart rate, b/min				
Non-adjusted	72.7	71.7	69.0	*< 0.001*
ANOVA adjusted for sex and age	72.6	71.8	69.1	*< 0.001*

* by chi-square or ANOVA

Standardized regression coefficient of heart rate over log-transformed activity at the baseline exam: -0.127 in single-variable regression and -0.111 in regression adjusted for sex and age (P < 0.001).

Table S4 – Tertiles of habitual alcohol intake at the baseline exam: number of examinees, median of intake, factor-specific score, gender prevalence, mean age, and mean of serum gammglutamyl-transferase used as the validation variable.

	Tertile of alcohol intake			*P for trend**
	1	2	3	
Tertiles thresholds, habitual alcohol intake at the baseline exam, g/d	< 2	2 – 23	> 23	
Number of individuals	1558	1289	1822	
Median alcohol intake, g/d	0	12	36	
Factor-specific score	2	1	0	
% men	23.0%	34.4%	71.7%	*<0.001*
Age, years	46.0	50.4	53.0	*<0.001*
Serum gammaglutamyl-transferase, U/L				
Non-adjusted	22.7	26.3	43.7	*<0.001*
ANOVA adjusted for sex and age	26.6	27.7	39.4	*<0.001*

* by chi-square or ANOVA

Standardized regression coefficient of serum gammaglutamyl-transferase over log-transformed alcohol intake at the baseline exam: 0.262 in single-variable regression and 0.174 in regression adjusted for sex and age ($P < 0.001$).

Table S5 – Tertiles of urea/creatinine ratio in overnight urine at the baseline exam: number of examinees, median of urinary urea, factor-specific score, gender prevalence, mean age, and mean of serum urea used as the validation variable.

	Tertile of urea/creatinine ratio			
	1	2	3	
Tertile thresholds of urea/creatinine ratio, g/g				
Men – age 18-24	< 11.8	11.9 – 14.8	> 14.8	
Men – age 25-34	< 11.3	11.4 – 14.1	> 14.1	
Men – age 35-44	< 12.0	12.1 – 15.6	> 15.6	
Men – age 45-54	< 12.4	12.5 – 15.6	> 15.6	
Men – age 55-64	< 13.1	13.2 – 16.9	> 16.9	
Men – age 65-74	< 13.3	13.4 – 17.5	> 17.5	
Men – age 75-84	< 13.1	13.2 – 17.1	> 17.1	
Men – age > 84	< 11.1	11.2 – 17.3	> 17.3	
Women – age 18-24	< 12.6	12.7 – 16.9	> 16.9	
Women – age 25-34	< 13.1	13.2 – 18.0	> 18.0	
Women – age 35-44	< 15.0	15.1 – 19.3	> 19.3	
Women – age 45-54	< 16.3	16.4 – 21.2	> 21.2	
Women – age 55-64	< 17.3	17.4 – 22.3	> 22.3	
Women – age 65-74	< 17.1	17.2 – 22.9	> 22.9	
Women – age 75-84	< 17.1	17.2 – 22.3	> 22.3	
Women – age > 84	< 14.8	14.9 – 20.3	> 20.3	
Number of examinees	1421	1827	1421	
Median of urea/creatinine ratio, g/g	11.2	15.9	22.7	*P for trend**
Factor-specific score	0	1	2	
% men	45.2%	45.1%	45.2%	*0.999*
Age, years	49.8	50.1	50.0	*0.725*
Serum urea, mmol/L				
Non-adjusted	5.50	6.00	6.17	*<0.001*
Adjusted for sex and age	5.71	6.06	6.34	*<0.001*

Tertiles were defined separately in men and women by age strata.

* by chi-square or ANOVA

Standardized regression coefficient of serum urea over log-transformed urinary urea/creatinine ratio at the baseline exam: 0.977 in single-variable regression and 0.976 in regression adjusted for sex and age (P < 0.001).

Table S6 – Tertiles of sodium/creatinine ratio in overnight urine at the baseline exam: number of examinees, median of urinary sodium, factor-specific score, gender prevalence, mean age, and mean of hematocrit used as the validation variable.

	Tertile of sodium/creatinine ratio			*P for trend**
	1	2	3	
Tertiles thresholds, sodium/creatinine ratio at the baseline exam, mmol/g				
Men				
age 18-24	< 50	50 – 79	> 79	
age 25-34	< 50	50 – 83	> 83	
age 35-44	< 65	65 – 99	> 99	
age 45-54	< 73	73 – 115	> 115	
age 55-64	< 94	94 – 143	> 143	
age 65-74	< 104	104 – 144	> 144	
age 75-84	< 106	106 – 162	> 162	
age > 84	< 103	103 – 158	> 158	
Women				
age 18-24	< 58	58 – 102	> 102	
age 25-34	< 71	71 – 107	> 107	
age 35-44	< 79	79 – 125	> 125	
age 45-54	< 95	95 – 142	> 142	
age 55-64	< 104	104 – 156	> 156	
age 65-74	< 118	118 – 178	> 178	
age 75-84	< 131	131 – 214	> 214	
age > 84	< 143	143 – 234	> 234	
Number of individuals	1502	1616	1551	
Median of sodium/creatinine ratio, mmol/g	59	107	176	
Factor-specific score	0	1	2	
% men	45.0%	44.9%	45.5%	*0.775*
Age, years	49.7	50.2	50.1	*0.561*
Hematocrit, %				
Non-adjusted	42.8	42.7	42.5	*0.023*
Adjusted for sex and age	42.9	42.7	42.5	*0.002*

Tertiles were defined separately in men and women by age strata.

* by chi-square or ANOVA

Standardized regression coefficient of hematocrit over log-transformed urinary sodium/creatinine ratio at the baseline exam: -0.125 in single-variable regression and -0.043 in regression adjusted for sex and age ($P \leq 0.001$).

Table S7 – Tertiles of potassium/creatinine ratio in overnight urine at the baseline exam: number of examinees, median of urinary potassium, factor-specific score, gender prevalence, mean age, and mean of serum potassium used as the validation variable.

	Tertile of potassium/creatinine ratio			
	1	2	3	*P for trend**
Number of individuals	1501	1639	1529	
Tertiles thresholds, potassium/creatinine ratio at the baseline exam, mmol/g				
Men				
age 18-24	< 13	13 – 19	> 19	
age 25-34	< 14	14 – 21	> 21	
age 35-44	< 17	17 – 24	> 24	
age 45-54	< 19	19 – 25	> 25	
age 55-64	< 22	22 – 31	> 31	
age 65-74	< 24	24 – 33	> 33	
age 75-84	< 24	24 – 36	> 36	
age > 84	< 22	22 – 41	> 41	
Women				
age 18-24	< 18	18 – 26	> 26	
age 25-34	< 19	19 – 27	> 27	
age 35-44	< 22	22 – 29	> 29	
age 45-54	< 23	23 – 32	> 32	
age 55-64	< 25	25 – 34	> 34	
age 65-74	< 28	28 – 38	> 38	
age 75-84	< 32	32 – 42	> 42	
age > 84	< 32	32 – 49	> 49	
Median of potassium/creatinine ratio, mmol/g	17.2	25.7	39.5	
Factor-specific score	2	1	0	
% men	44.6%	45.9%	44.9%	*0.845*
Age, years	49.1	50.3	50.4	*0.144*
Serum potassium, mmol/L				
Non-adjusted	4.10	4.13	4.18	*<0.001*
Adjusted for sex and age	4.10	4.13	4.17	*<0.001*

Tertiles were defined separately in men and women by age strata.

* by chi-square or ANOVA

Standardized regression coefficient of serum potassium over log-transformed urinary potassium/creatinine ratio at the baseline exam: 0.106 in single-variable regression and 0.089 in regression adjusted for sex and age ($P < 0.001$).

Table S8 – Consistency over time of the habitual physical activity in leisure time: median of activity at the baseline exam and at the follow-up exam by tertile of activity at the baseline exam.

	Tertile of physical activity at the baseline exam			*P for trend**
	1	2	3	
Number of examinees with complete data at baseline exam and follow-up exam	1230	993	747	
Median of habitual physical activity in leisure time at the baseline exam, min/d	0	12	43	*< 0.001*
Median of habitual physical activity in leisure time at the follow-up exam, min/d	15	20	30	*< 0.001*

* by ANOVA

Standardized regression coefficient of the log-transformed physical activity at the follow-up exam over the log-transformed physical activity at the baseline exam: 0.139 in single-variable regression and 0.114 in regression adjusted for sex and age (P<0.001).

Table S9 – Consistency over time of the habitual alcohol intake: median of intake at the baseline exam and the follow-up exam by tertile of intake at the baseline exam.

	Tertile of alcohol intake at the baseline exam			*P for trend*
	1	2	3	
Number of examinees with complete data at the baseline exam and follow-up exam	1046	819	1169	
Median of habitual alcohol intake at the baseline exam, g/d	0	12	36	*< 0.001*
Median of habitual alcohol intake at the follow-up exam, g/d	0	4	24	*<0.001*

* by ANOVA

Standardized regression coefficient of the log-transformed alcohol intake at the follow-up exam over the log-transformed alcohol intake at the baseline exam: 0.629 in single-variable regression and 0.513 in regression adjusted for sex and age (P<0.001).

Table S10 – Consistency of urinary urea/creatinine ratio over time and over different types of urine collections: median of the urea/creatinine ratio in the overnight urine collection of the baseline exam and in the 24-hour urine collection of the exam of the Intersalt study by tertile of urea/creatinine ratio at the baseline exam§.

	Tertile of urea/creatinine ratio at the baseline exam§			*P for trend**
	1	2	3	
Number of examinees with complete data at the baseline exam and at the exam of the Intersalt study	54	56	48	
Urinary urea/creatinine ratio in the overnight collection of the baseline exam	12.0	15.2	21.9	*< 0.001*
Urinary urea/creatinine ratio in the 24-hour collection of the exam of the Intersalt Study	14.8	16.0	18.3	*< 0.001*

Date of the exam of the Intersalt study: 1983-86
Date of the baseline exam: 1989-92

§Tertiles were defined separately in men and women for the following strata of age: 18-24, 25-34, 35-44, 45-54, 55-64, 65-74, 75-84, and ≥ 85 years.

* by ANOVA

Standardized regression coefficient of the log-transformed urinary urea/creatinine ratio in the 24-hour urine collection of the Intersalt study over the log-transformed urinary urea/creatinine ratio in the overnight urine collection of the baseline exam: 0.239 in single-variable regression and 0.230 in regression adjusted for sex and age (P < 0.001).

Table S11 – Consistency of urinary sodium/creatinine ratio over time and over different types of urine collections: median of the sodium/creatinine ratio in the overnight urine collection of the baseline exam and in the 24-hour urine collection of the exam of the Intersalt study by tertile of sodium/creatinine ratio at the baseline exam[§].

	Tertile of sodium/creatinine ratio at the baseline exam[§]			*P for trend**
	1	2	3	
Number of examinees with complete data at the baseline exam and at the exam of the Intersalt study	57	54	57	
Urinary sodium/creatinine ratio in the overnight urine collection of the baseline exam	59	96	169	*< 0.001*
Urinary sodium/creatinine ratio in 24-hour urine collection of the exam of the Intersalt Study	105	129	154	*< 0.001*

Date of the exam of the Intersalt Study: 1983-86
Date of the baseline exam: 1989-92

[§]Tertiles were defined separately in men and women for the following strata of age: 18-24, 25-34, 35-44, 45-54, 55-64, 65-74, 75-84, and ≥ 85 years.

* by ANOVA

Standardized regression coefficient of the log-transformed urinary sodium/creatinine ratio in the 24-hour urine collection of the Intersalt study over the log-transformed urinary sodium/creatinine ratio in the overnight urine collection of the baseline exam: 0.212 in single-variable regression and 0.158 in regression adjusted for sex and age (P<0.05).

Table S12 – Consistency of urinary potassium over time and over different types of urine collections: median of the potassium/creatinine ratio in the overnight urine collection of the baseline exam and in the 24-hour urine collection of the exam of the Intersalt study by tertile of potassium/creatinine ratio at the baseline exam[§].

	Tertile of potassium/creatinine ratio at the baseline exam[§]			*P for trend**
	1	2	3	
Number of examinees with complete data at the baseline exam and at the exam of the Intersalt study	57	61	50	
Urinary potassium/creatinine ratio in the overnight urine collection of the baseline exam	16	24	35	*< 0.001*
Urinary potassium/creatinine ratio in the 24-hour urine collection of exam of the Intersalt Study	31	44	54	*< 0.001*

Date of the exam of the Intersalt Study: 1983-86
Date of the baseline exam: 1989-92

[§]Tertiles were defined separately in men and women for the following strata of age: 18-24, 25-34, 35-44, 45-54, 55-64, 65-74, 75-84, and ≥ 85 years.

* by ANOVA

Standardized regression coefficient of the log-transformed urinary potassium/creatinine ratio in the 24-hour urine collection of the Intersalt study over the log-transformed urinary potassium/creatinine ratio in the overnight urine collection of the baseline exam: 0.275 in single-variable regression and 0.160 in regression adjusted for sex and age (P<0.04).

Figure S1 - Frequency distribution of the cumulative score in the 4669 examinees with baseline data. Upper panel: distribution before merging the low-n tails (skewness = 0.035, mean±SD = 5±2); lower panel: distribution after merging score 0 into score 1 and score 10 into score 9 (skewness = 0.016, mean±SD = 5±2).

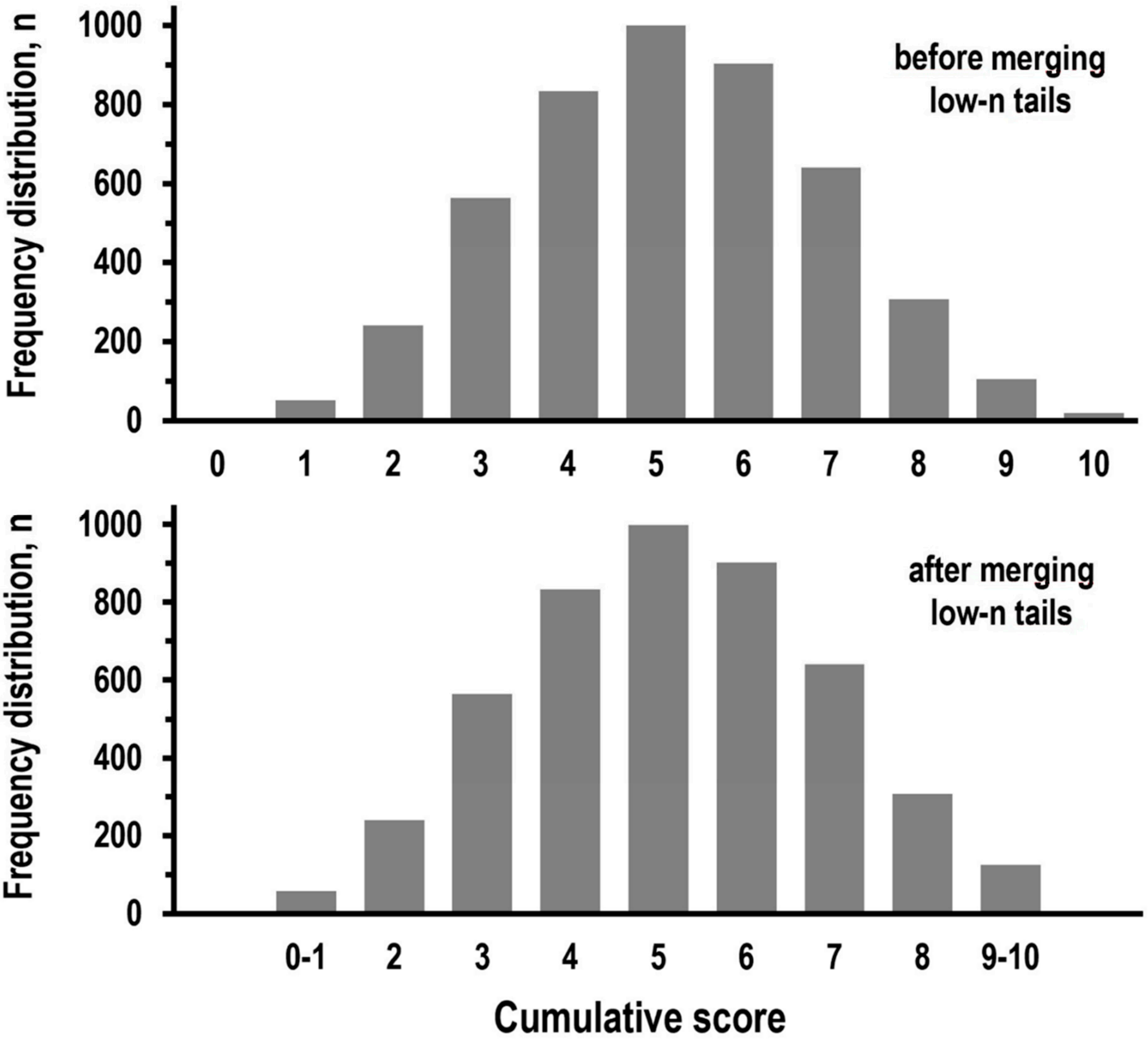

Figure S2 - Frequency distribution of the cumulative score in the examinees with complete baseline data and follow-up data (upper panel, skewness = 0.019, mean±SD = 5±2) and in the examinees with complete baseline data and follow-up data and without low kidney function at baseline (lower panel, skewness = 0.022, mean±SD = 5±2).

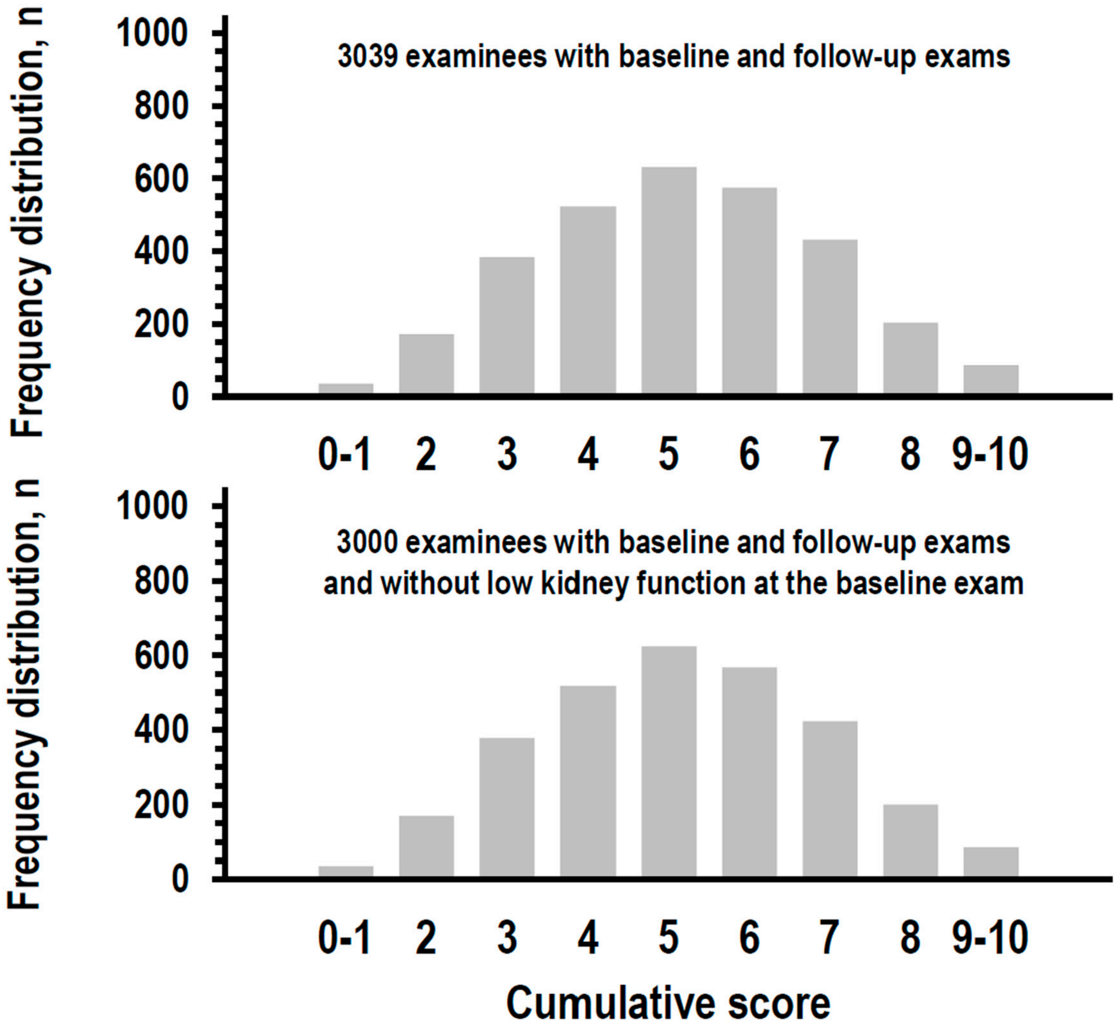

Table S13 – Baseline mans by the cumulative score of the variable used as proxy of the modifiable factors.

Score	N examinees	Heart rate, b/min	Serum gamma-glutamyl-transferase, U/L	Serum urea mmol/L	Hematocrit, %	Serum potassium, mmol/L
0-1	58	71.1	73.8	5.76	45.0	4.18
2	240	70.9	46.6	5.90	44.3	4.17
3	564	70.7	36.2	5.85	43.8	4.17
4	834	70.6	35.2	5.96	43.4	4.13
5	999	71.2	30.5	6.09	42.8	4.13
6	903	72.4	28.3	6.07	41.9	4.12
7	641	72.2	27.7	6.23	41.8	4.15
8	306	72.1	23.0	6.07	41.3	4.10
9-10	124	73.4	23.9	6.13	41.6	4.11
**P for trend along the cumulative score*		*< 0.001*	*< 0.001*	*< 0.001*	*< 0.001*	*0.010*

* by ANOVA
